# Solubilization of struvite and biorecovery of cerium by *Aspergillus niger*

**DOI:** 10.1007/s00253-021-11721-0

**Published:** 2022-01-04

**Authors:** Xia Kang, Laszlo Csetenyi, Xiang Gao, Geoffrey Michael Gadd

**Affiliations:** 1 Geomicrobiology Group, School of Life Sciences, University of Dundee, Dundee, DD1 5EH UK Scotland; 2 Key Laboratory of Environmental and Applied Microbiology, CAS; Environmental Microbiology Key Laboratory of Sichuan Province, Chengdu Institute of Biology, Chinese Academy of Sciences, Chengdu, 610041 China; 3 Concrete Technology Group, Department of Civil Engineering, University of Dundee, Dundee,, DD1 4HN UK Scotland; 4 School of Chemistry, University of St Andrews, St Andrews, KY16 9ST Scotland, UK; 5 State Key Laboratory of Heavy Oil Processing, Beijing Key Laboratory of Oil and Gas Pollution Control, College of Chemical Engineering and Environment, China University of Petroleum, 18 Fuxue Road, Changping District, Beijing, 102249 China

**Keywords:** *Aspergillus niger*, Rare earth elements, Struvite, Cerium, Phosphate

## Abstract

**Abstract:**

Cerium has many modern applications such as in renewable energies and the biosynthesis of nanomaterials. In this research, natural struvite was solubilized by *Aspergillus niger* and the biomass-free struvite leachate was investigated for its ability to recover cerium. It was shown that struvite was completed solubilized following 2 weeks of fungal growth, which released inorganic phosphate (P_i_) from the mineral by the production of oxalic acid. Scanning electron microscopy (SEM) showed that crystals with distinctive morphologies were formed in the natural struvite leachate after mixing with Ce^3+^. Energy-dispersive X-ray analysis (EDXA), X-ray diffraction (XRD) and Fourier-transform infrared spectroscopy (FTIR) confirmed the formation of cerium phosphate hydrate [Ce(PO_4_)·H_2_O] at lower Ce concentrations and a mixture of phosphate and cerium oxalate decahydrate [Ce_2_(C_2_O_4_)_3_·10H_2_O] at higher Ce concentrations. The formation of these biogenic Ce minerals leads to the removal of > 99% Ce from solution. Thermal decomposition experiments showed that the biogenic Ce phosphates could be transformed into a mixture of CePO_4_ and CeO_2_ (cerianite) after heat treatment at 1000 °C. These results provide a new perspective of the fungal biotransformation of soluble REE species using struvite leachate, and also indicate the potential of using the recovered REE as biomaterial precursors with possible applications in the biosynthesis of novel nanomaterials, elemental recycling and biorecovery.

**Key points:**

• *Cerium was recovered using a struvite leachate produced by *A. niger*.*

• *Oxalic acid played a major role in struvite solubilization and Ce phosphate biorecovery.*

• *Resulting nanoscale mineral products could serve as a precursor for Ce oxide synthesis.*

## Introduction

Cerium (Ce) is classified as one of the light rare earth elements (REE) in the lanthanide series and bears similar physical and chemical properties to lanthanum (Massari and Ruberti 2013). The predominant source of cerium is monazite ore [(Ce, La, Nd)PO_4_], which contains 40–50% cerium on average (Kumari et al. 2015). It is estimated that by the year 2030, the global annual production of Ce will be 186.54 kilotonnes, which is the highest among all the other REE and accounts for 40.5% of total REE production (Moss et al. 2013). Cerium compounds are traditionally used in components of cigarette lighter flints, petroleum cracking catalysts, energy-efficient light sources and solid oxide fuel cells (SOFC) (Li et al. 2000; van Krevel et al. 2002; Martínez-Arias et al. 2005; Haque et al. 2014). More recently, nanoscale cerium compounds have been synthesized and new applications discovered in biomedical sciences such as bioimaging, biosensing and therapeutic nanomaterials (Babu et al. 2010; Siposova et al. 2019; Singh et al. 2020). Therefore, as more novel applications are emerging, cerium will be one of the most promising rare earth elements that deserves attention.

Biomineralization refers to the biologically mediated formation of new minerals and is an indispensable step in geomicrobial processes (Verrecchia et al. 1993; Adeyemi and Gadd 2005; Gadd 2010, 2017). It is well known that biomineralization can occur as a result of interactions between microbes and metal species in both solid and aqueous forms (Francis 1998; Rawlings et al. 2003). Microbial biomineralization has been taken advantage of in recent years because of its environmentally friendly nature and cost-effectiveness, and therefore has potential applications for the removal of environmental pollutants and the recovery of precious metals from wastes (Liang and Gadd 2017; Yang et al. 2019; Li et al. 2020a,b; Xu et al. 2020). Geoactive fungi play an important role in the biomineralization of a broad range of minerals and have been investigated for their potential in the recycling of elements and biosynthesis of new biomaterials (Gadd 2007; Liang and Gadd 2017; Kang et al. 2020, 2021). The fundamental mechanisms behind fungal biomineralization involve the production of metabolites such as oxalate and carbon dioxide, or the release of ligands like carbonates and phosphates from organic and inorganic sources, as well as proteins and enzymes that can directly interact with the metal/mineral (Gadd 2007, 2010; Li et al. 2014; Kumari et al. 2016; Dhami et al. 2017; Fomina et al. 2007; Liu et al. 2019; Kirtzel et al. 2020; Kang et al. 2020, 2021; Mendes et al. 2021a,b; Liu et al. 2021). 

Phosphates play an important role in the biogeochemical cycles of elements. Common P-bearing minerals such as phosphate rocks and apatites (Ca-phosphates) can be solubilized by phosphate-solubilizing microorganisms (Bolan et al. 1994; Zhenghua et al. 2001; Mendes et al. 2021a,b). Some fungal species, e.g. *Aspergillus niger* and *Paecilomyces javanicus*, were able to precipitate soluble U(IV) and Pb when supplemented with an organic P source (glycerol-2-phosphate), suggesting that this process could be exploited for biorecovery and bioremediation (Liang et al. 2015, 2016a, b; Rhee et al. 2016). Struvite (MgNH_4_PO_4_·6H_2_O) is commonly found in sewage treatment plants and sludge handling facilities, causing blockages to pipelines (Doyle and Parsons 2002; Wang et al. 2018). A large amount of research has concentrated on the recovery of P and Mg by struvite crystallization (Le Corre et al. 2009; Liang et al. 2019). In contrast, few studies have ventured the idea of solubilizing struvite and recycling P and Mg as other useful materials through microbial processes (Hernández Jiménez et al. 2020; Kecskésová et al. 2020). The solubility product of struvite is 10^−13.29^ < *K*_sp_ < 10^−13.08^, which is much greater than that of calcium phosphate (*K*_sp_ = 1.3 × 10^−26^), and it has been confirmed that acid-producing *A. niger* is able to solubilize both natural and synthetic struvite releasing high amounts of P into the leachate (Hanhoun et al. 2011; Suyamud et al. 2020; Ferrier et al. 2021). Cerium phosphate nanotubes and nanowires can be synthesized, using complex chemical methods, which exhibit optical, photoprotective and other properties (Bu et al. 2004; Tang et al. 2005; Xing et al. 2006; de Lima and Serra 2013). However, there is a lack of research on the biosynthesis of this potentially useful material through a microbial process.

The present study offers insights into and provides fundamental knowledge about the interactions between *A. niger* and cerium with an aim to explore novel approaches for biorecovery of REE as phosphate biominerals using a biomass-free struvite leachate. The work also contributes to further understanding of the extracellular synthesis of biomaterials from a fungal system.

## Methods and materials

### Microorganism and culture medium

A wild-type strain of *Aspergillus niger* (ATCC 1015), which was routinely maintained on malt extract agar (MEA) (Lab M Limited, Bury, UK) in the dark at 25 °C, was used in the present study. Modified Czapek-Dox medium (MCD) consisted of (1^−1^ Milli-Q water) D-glucose 30 g, NaNO_3_ 2 g, Na_2_HPO_4_ 1 g, MgSO_4_·7H_2_O 0.5 g, KCl 0.5 g and FeSO_4_·7H_2_O 0.01 g. The final pH was adjusted to pH 5.5 with sterile 1 M HCl prior to autoclaving for 15 min at 115 °C.

### Solubilization of struvite

A natural struvite sample was used to provide P-rich biomass-free spent culture medium for the precipitation of Ce phosphate. Struvite fragments were obtained from the operating site (Exeter, UK) of Veolia Water Outsourcing (London, UK) and pulverised using a pestle and mortar. *A. niger* was initially inoculated on MEA plates and grown for several days until a luxuriant growth state was reached. A spore suspension was then made using the following procedure: sterile 0.1% (v/v) Tween 80 was poured on the fungal colony, mixed well by pipetting multiple times, filtered through micro-pored (40 μm) sterile muslin cloth (Henry Simon Limited, Cheshire, UK) and washed three times using sterile Milli-Q water by centrifugation (2553 g, 30 min). The number of spores in the suspension was determined using a Neubauer-improved counting chamber (Paul Marienfeld GmbH & Co. KG, Lauda-Königshofen, Germany). The suspension was diluted to desired concentration using sterile Milli-Q water before use. The struvite sample was oven-sterilized at 105 °C for 48 h and added at 1% (w/v) to 500 ml Erlenmeyer flasks containing 200 ml MCD medium, which were inoculated with an appropriate amount of *A. niger* spore suspension resulting in 1 × 10^6^ spores ml^−1^ final concentration. Flasks without inoculation served as the control. All the flasks were maintained in a shaking incubator (GE Healthcare, Buckinghamshire, UK) for 14 days in the dark (125 rpm, 25 °C). An aliquot of approximately 0.5 ml culture supernatant was taken every other day for pH measurement using a flat-tipped pH probe (VWR International, Lutterworth, England, UK) and other analyses.

### Biorecovery of Ce phosphate

The biomass-free spent liquid culture medium was obtained by vacuum pump-filtration through a Whatman filter paper (GE Healthcare, Buckinghamshire, UK). Metal cations in the filtered medium were removed using AG® 50 W-X8 cation exchange resin (20–50 mesh, in hydrogen form) (Bio-Rad Laboratories, CA, USA) which efficiently replaces all metal ions with H^+^. This was achieved by adding 0.5 g resin into 5 ml biomass-free culture medium and maintaining on a roller mixer (Stuart Equipment, Stone, Staffordshire, UK) for 24 h at ambient temperature. After metal cations were removed, the pH of the biomass-free culture medium was adjusted to pH 7.5 using 4 M NaOH and filtered again using a 0.25 μm Minisart syringe filter (Sartorius Stedim Biotech GmbH, Göttingen, Germany) before use. Biorecovery experiments were performed using a 10-ml reaction system in 15 ml centrifuge tubes by mixing biomass-free liquid culture medium with sterile CeCl_3_ at the following final concentrations: 5, 20, 40 and 50 mM Ce^3+^, and incubating on a roller mixer (Stuart Equipment, Stone, Staffordshire, UK) for 24 h at room temperature. The phosphate biominerals were collected and washed three times using Milli-Q water, and the supernatant pH was measured. Each treatment was performed in triplicate.

### Harvest, desiccation and weighing of biominerals

The biominerals were harvested and washed three times by centrifugation (2553 g, 30 min) using a J6-MI high-capacity centrifuge (Beckman Coulter, High Wycombe, UK). All biominerals were dried for at least 2 weeks at room temperature in a desiccator filled with Chameleon silica gel (VWR International Ltd., Lutterworth, England, UK). The yield of biominerals was measured by weighing using a Galaxy HR-150A analytical balance (A&D Company, Tokyo, Japan).

### Determination of cerium

Ce concentrations in supernatants after liquid reactions were measured using the Arsenazo III colorimetric method (Hogendoorn et al. 2018). This was achieved by mixing 1 ml of an appropriately diluted sample with 1 ml 0.02% (w/v) Arsenazo III solution (Sigma-Aldrich, St. Louis, USA) and 8 ml pH 2.8 potassium hydrogen phthalate buffer. After 10 min, the OD_658 nm_ of mixture was measured using an Ultrospec 2100 pro spectrophotometer (Biochrom Ltd., Holliston, MA, USA). Ce concentrations in the liquid samples were calculated according to a standard curve created using the following concentrations: 0, 0.1, 0.2, 0.3, 0.5 and 1.0 mg l^−1^ REE.

### Determination of oxalic acid

The determination of oxalic acid in the culture supernatant was carried out using an UltiMate 3000 high-performance liquid chromatography (HPLC) system (Dionex, San Diego, CA, USA) fitted with an Aminex HPX-87H column in connection with a Micro-Guard cation H^+^ refill cartridge (Bio-Rad Laboratories, CA, USA). Samples were prepared by appropriately diluting the original liquid. Standard solutions contained the following acids: 5, 10, 15 and 20 mM citric acid (Sigma-Aldrich, St. Louis, USA); 1, 2, 3 and 4 mM oxalic acid (Sigma-Aldrich, St. Louis, USA). All samples and standards were filtered through a 0.25-μm Minisart syringe filter (Sartorius Stedim Biotech GmbH, Göttingen, Germany) and pipetted into HPLC certified vials (Sigma-Aldrich, St. Louis, USA). The mobile phase was 5 mM H_2_SO_4_, which was prepared using HPLC LiChropur 98% sulfuric acid (Merck KGaA, Darmstadt, Germany) and degassed by pump-filtration through a 0.45-μm Whatman cellulose nitrate membrane filter (GE Healthcare, Buckinghamshire, UK). The parameters for the HPLC system were as follows: flow rate at 0.6 ml min^−1^, column temperature at 35℃, sample injection volume 20 μl and wavelength of the UV detector 210 nm. The concentration of organic acids was automatically calculated by the Chromeleon 6.8 Chromatography Data System Software (Thermo Fisher Scientific, MA, USA) at default settings. The measurements were performed in at least three replicates.

### EDXA, SEM, XRD, XRF, TGA and FTIR

Dried mineral samples were mounted on adhesive carbon tape on 25 mm × 5 mm aluminium electron microscopy stubs (Agar Scientific Ltd., Essex, UK) before being examined using an energy-dispersive X-ray spectroscopy analysis (EDXA) system (Oxford Inca, Abingdon, Oxon, UK) operating in conjunction with a Jeol JSM-7400F field emission scanning electron microscope (JEOL Ltd., Tokyo, Japan) at an accelerating voltage of 15 kV for 100 s. Samples for scanning electron microscopy (SEM) were coated with a layer of 10 nm gold and platinum using a Cressington 208HR sputter coater (Ted Pella, Redding, CA, USA) prior to examination using a field emission scanning electron microscope (Jeol JSM7400F) operating at an accelerating voltage of 5 kV. X-ray diffraction (XRD) was performed using a Hiltonbrooks X-ray diffractometer (HiltonBrooks Ltd., Crewe, UK) furnished with a monochromatic CuKα source and curved graphite, single-crystal chronometer (30 mA, 40 kV). To prepare for XRD, samples were ground to a fine powder using a ceramic mortar and pestle and compacted tightly on the reverse side of an aluminium specimen holder (15 × 20 × 2 mm) held against a glass slide. The back cover was then snapped into place and the glass slide was removed from the holder. Duplicate samples were analyzed over the range 3–60° 2θ at a scan rate of one degree min^−1^ in 0.1 degree increments. X-ray fluorescence (XRF) spectroscopy was carried out using a Philips Zetium PW5400 sequential spectrometer with an RhKα source (Malvern Panalytical, Malvern, UK), and calibrated with certified standard materials. The samples were placed in a 32-mm-diameter pellet mould, and were compacted under loads of 75 kN for 5 min and 150 kN for a further 10 min and then transferred to a specimen cup that had a 27-mm-diameter viewing aperture. The results are expressed as oxides. Thermal decomposition of the biominerals for thermogravimetric analysis (TGA) was carried out using a NETZSCH STA 409PC TG/DTG/DTA analyser fitted with a SiC furnace (NETZSCH Group, Selb, Germany). Small amounts (< 100 mg) of samples were heated to 1000 °C at a heating rate of 10 K min^−1^ using dry N_2_ as a purge gas at a flow rate of 100 cm^3^ min^−1^ and maintained at 1000 °C until constant weight. Fourier-transform infrared spectroscopy (FTIR) was employed for further accurate identification of nanoscale biomineral samples. Dried samples were ground to a fine powder using a pestle and mortar for the analysis, which was carried out using an IRAffinity-1S compact Fourier-transform infrared spectrophotometer (Shimadzu Corporation, Kyoto, Japan). The wavenumber was measured at a range of 400–4000 cm^−1^ (wavelengths 2.5 to 25 µm) at a resolution of 1 cm^−1^.

## Results

### Mineral profile and solubilization of struvite

**Fig. 1:**
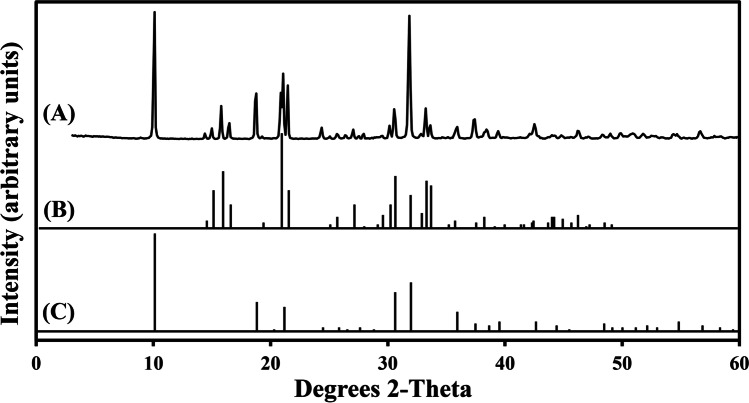
XRD patterns of (**A**) natural struvite. Shown below the experimental patterns are (**B**) reference pattern of the standard mineral NH_4_MgPO_4_·6H_2_O (card number: 15–762) and (**C**) reference pattern of standard NH_4_MgPO_4_·H_2_O (card number: 20–663) from the Powder Diffraction File (PDF) database. Typical patterns are shown from one of several determinations

XRF showed that the natural struvite sample was mainly composed of P (48.782% P_2_O_5_) and Mg (24.711% MgO) (Table 1). Most of the other elements detected existed in trace amounts at < 1%. XRD analysis of the struvite samples used in these experiments showed that the natural struvite pattern (Fig. 1A) matched with the reference minerals of struvite (NH_4_MgPO_4_·6H_2_O) (PDF card number: 15–762) (Fig. 1B) and dittmarite (NH_4_MgPO_4_·H_2_O) (PDF card number: 20–663) (Fig. 1C).

**Table 1:** Elemental composition (% by mass) of the struvite sample determined using X-ray fluorescence

Element*	Natural struvite
Fe_2_O_3_	0.154
CaO	1.557
K_2_O	0.173
P_2_O_5_	48.782
MgO	24.711
Al_2_O_3_	0.123
Cl	0.012
SiO_2_	0.214
SO_3_	0.071
MnO	0.141
Co	0.007
Sum	75.970

^*^Some elements are expressed as oxides

Typical values are from several measurements

**Fig. 2:**
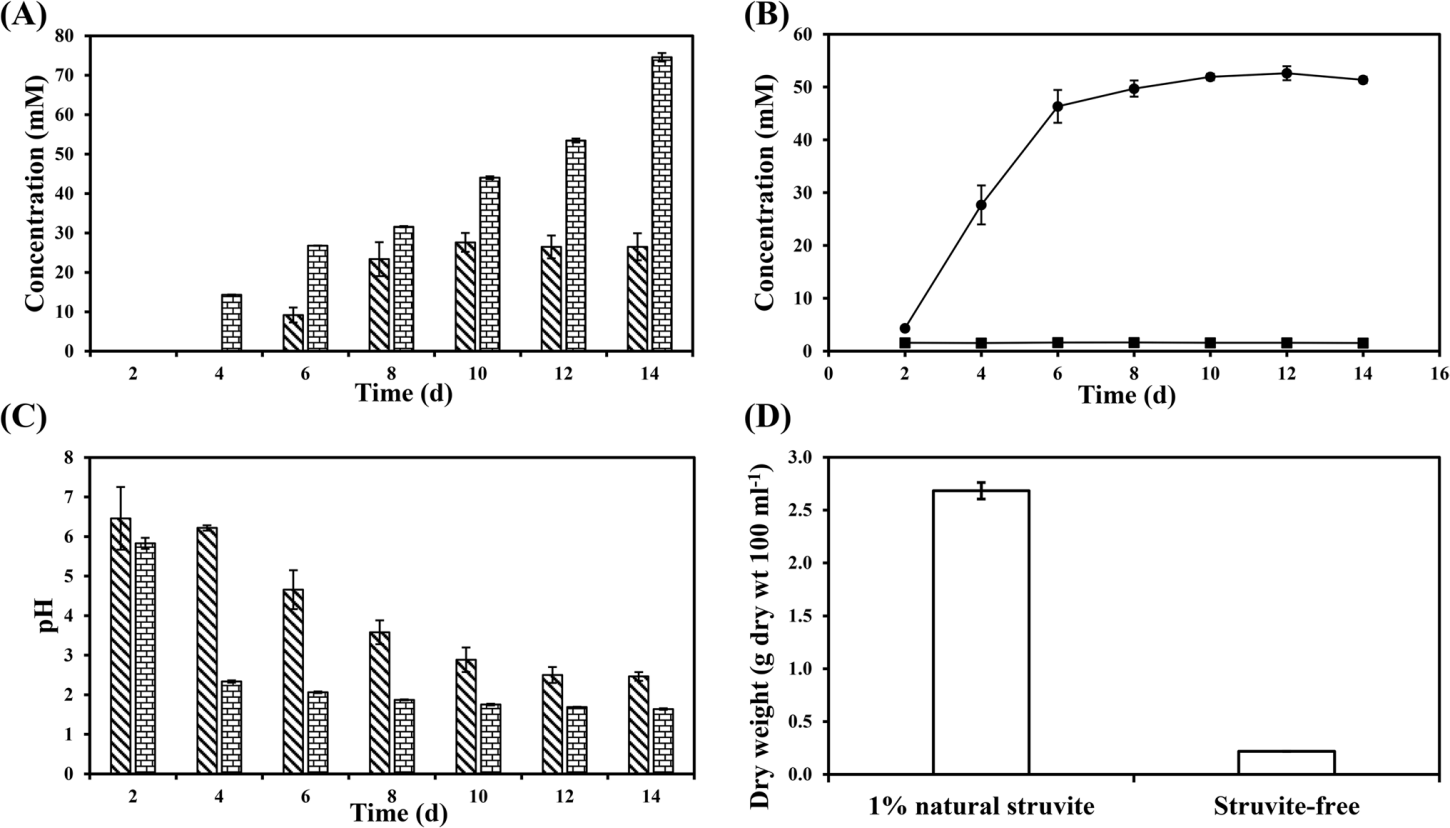
(**A**) Oxalic acid concentration and (**C**) supernatant pH in liquid MCD media inoculated with *A. niger* and supplemented with (diagonal-patterned bar) 1% natural struvite and (brick-patterned bar) without struvite during 14-day shake incubation at 25 °C and 125 rpm in the dark. (**B**) P_i_ concentration in liquid MCD media inoculated with *A. niger* and supplemented with (solid/black circle) 1% natural struvite and (solid/black square) without struvite under the same conditions. (**D**) Biomass yield of *A. niger* after 14-day incubation under the same conditions. Data are averages of at least three replicates and error bars show the standard error of the mean

During 14-day leaching of 1% (w/v) struvite-containing samples using *A. niger* in liquid MCD medium, oxalic acid, medium pH and inorganic phosphate (P_i_) concentration were measured. After the sixth day of incubation, oxalic acid was detected in all liquid samples at concentrations of 9.18 ± 1.90 mM and 26.77 ± 0.04 mM for the natural struvite leachate and struvite-free culture, respectively (Fig. 2A). The concentration of oxalic acid in both supernatants followed an increasing trend until the 14^th^ day at 26.49 ± 3.43 mM (natural struvite leachate) and 74.57 ± 1.07 mM (struvite-free control). A significant difference in the oxalic acid concentration was observed between the struvite leachate and the control, and the struvite leachate generally contained 50% lower amounts of oxalic acid than the control. As dissolution of struvite occurred, the release of soluble phosphate was detected on the 2^nd^ day of incubation (Fig. 2B). The concentration of soluble P_i_ increased, reached a plateau on the 6^th^ day and ended at 51.37 ± 0.59 mM for the natural struvite leachate. The medium pH showed a constant declining trend throughout the incubation period and ended at pH 2.46 ± 0.11 and pH 1.64 ± 0.02 for the natural struvite leachate and the struvite-free control, respectively (Fig. 2C). The medium pH of the struvite leachate was always significantly higher than the struvite-free control. Upon completion of the leaching process, the *A. niger* biomass yield was 2.68 ± 0.08 g 100 ml^−1^ (dry wt) and 0.22 ± 0.00 g 100 ml^−1^ (dry wt) for the natural struvite leachate and struvite-free control, respectively (Fig. 2D). An apparent difference was found between these two samples. An examination of the fungal pellets revealed that MCD medium supplemented with natural struvite led to more exuberant growth of *A. niger* than the struvite-free control.

### Cerium biorecovery using struvite leachate

**Fig. 3:**
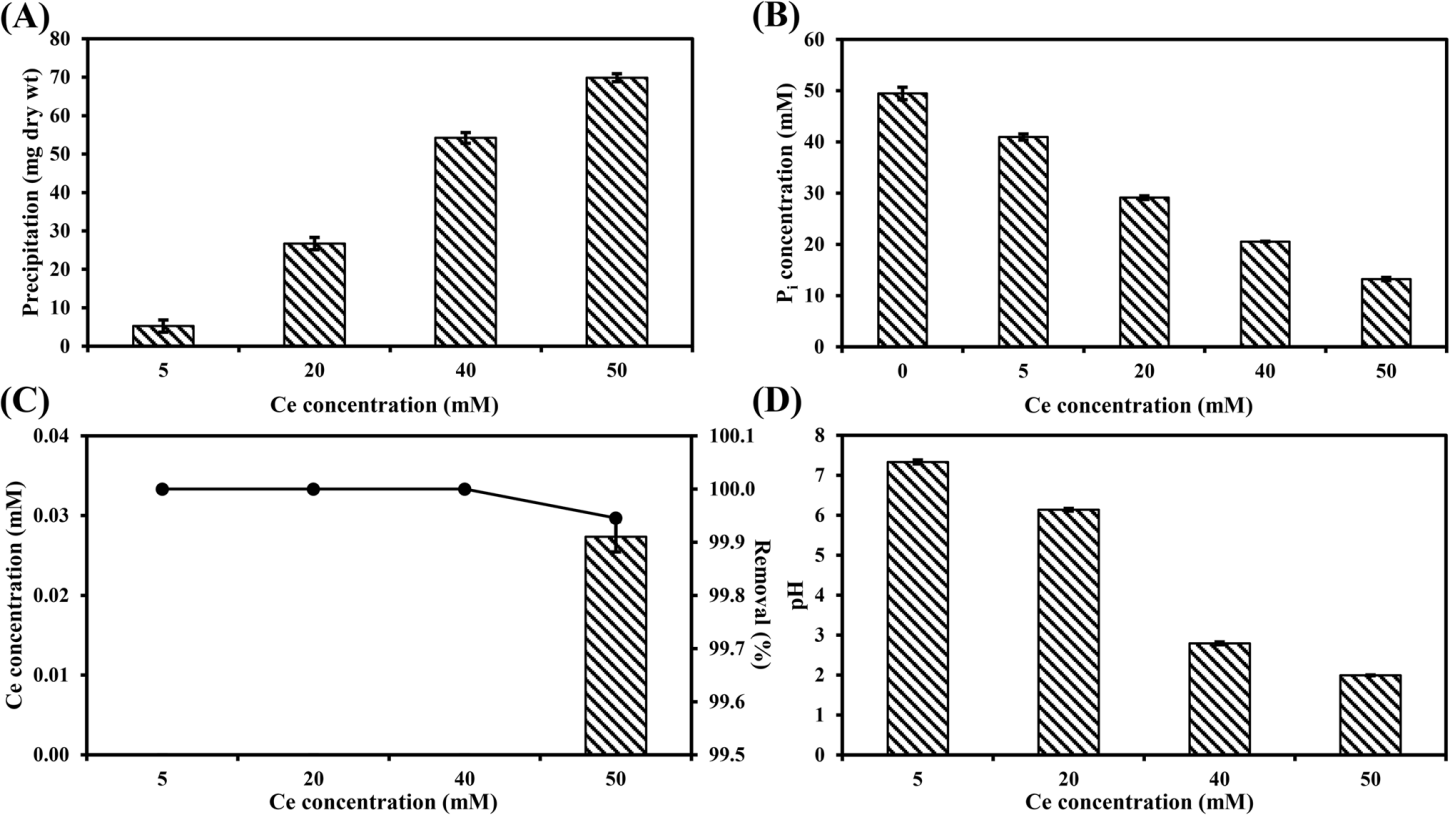
(**A**) Biomineral yield, (**B**) supernatant P_i_ concentration, (**C**) supernatant Ce concentration and (**D**) supernatant pH after reaction of 5 mM, 20 mM, 40 mM and 50 mM CeCl_3_ (final concentration) with natural struvite leachate for 24 h at room temperature on a roller mixer. The leachate was obtained from a 14-day shake incubation of *A. niger* in liquid MCD medium supplemented with 1% natural struvite. Solid/black circle: % removal of Ce^3+^ by 1% natural struvite leachate. Data are averages of at least three replicates and error bars show the standard error of the mean. Error bars are not shown when smaller than the dimensions of the symbols

The filtered and pH-adjusted struvite leachate was used to recover Ce from CeCl_3_ (final concentrations of 5, 20, 40 and 50 mM) and this resulted in a white precipitate after mixing. The amount of biominerals gradually increased as CeCl_3_ of higher concentration was added to the leachate of natural struvite (Fig. 3A). The total concentration of soluble P_i_ in the struvite leachate was significantly reduced as the concentration of CeCl_3_ increased (Fig. 3B). The concentration of P_i_ left in the leachate after mixing 50 mM CeCl_3_ with the struvite leachate was 13.22 ± 0.31 mM. Ce^3+^ was completely removed (100%) from the leachate of natural struvite after mixing with 5, 20 and 40 mM CeCl_3_, while 99.95% Ce^3+^ was removed after mixing with 50 mM CeCl_3_ (Fig. 3C). The amount of Ce^3+^ left after reaction with 50 mM CeCl_3_ was only 27.34 ± 1.91 μM in the natural struvite leachate. A significant drop of supernatant pH with the increasing amount of added CeCl_3_ was recorded for the leachate (Fig. 3D).

### Morphologies and identification of biominerals

**Fig. 4:**
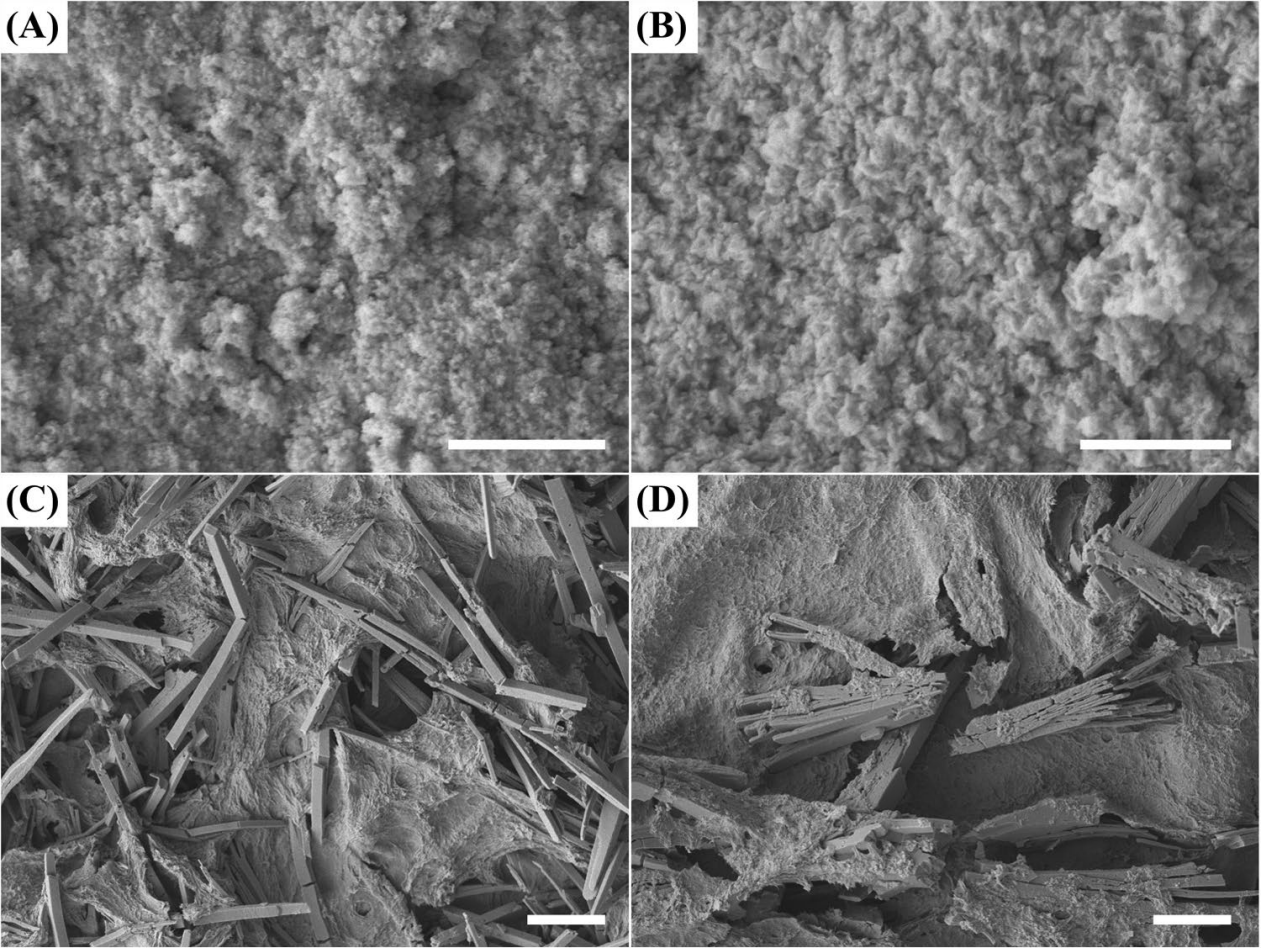
SEM images of biominerals formed after the reaction of natural struvite leachate with (**A**) 5 mM, (**B**) 20 mM, (**C**) 40 mM and (**D**) 50 mM CeCl_3_ (final concentration) for 24 h at room temperature. Scale bars: (A and B) 1 μm, (C and D) 10 μm. Typical images are shown from several similar examinations

**Fig. 5:**
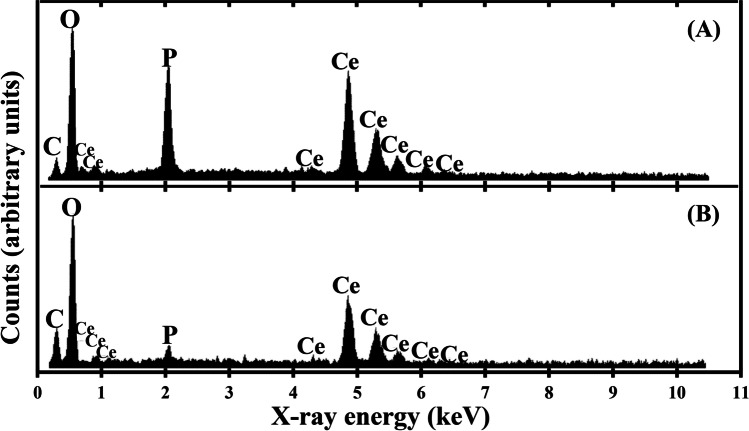
EDXA of (**A**) amorphous biominerals and (**B**) crystalline biominerals resulting from reaction of natural struvite leachate with different concentrations of CeCl_3_. Typical spectra are shown from several similar determinations

SEM examination showed that the biominerals formed after mixing the struvite leachate with 5 and 20 mM CeCl_3_ were aggregates of nanoparticles with no distinguishable boundaries (Fig. 4A and B). Slender cuboid crystals measuring ~ 20 μm long and ~ 2 μm wide along with the amorphous biominerals were observed in the precipitate formed in the natural struvite leachate after mixing with 40 mM CeCl_3_ (Fig. 4C). Large aggregates of tabloid crystals of similar size were precipitated after reaction of the natural struvite leachate with 50 mM CeCl_3_ (Fig. 4D). EDXA revealed that the amorphous biominerals formed at all concentrations of CeCl_3_ in the struvite leachate consisted of C, O, Ce and high amounts of P (Fig. 5A), whereas the crystals were composed of very low amounts of P as well as similar levels of the other elements as in the amorphous biominerals (Fig. 5B).

**Fig. 6:**
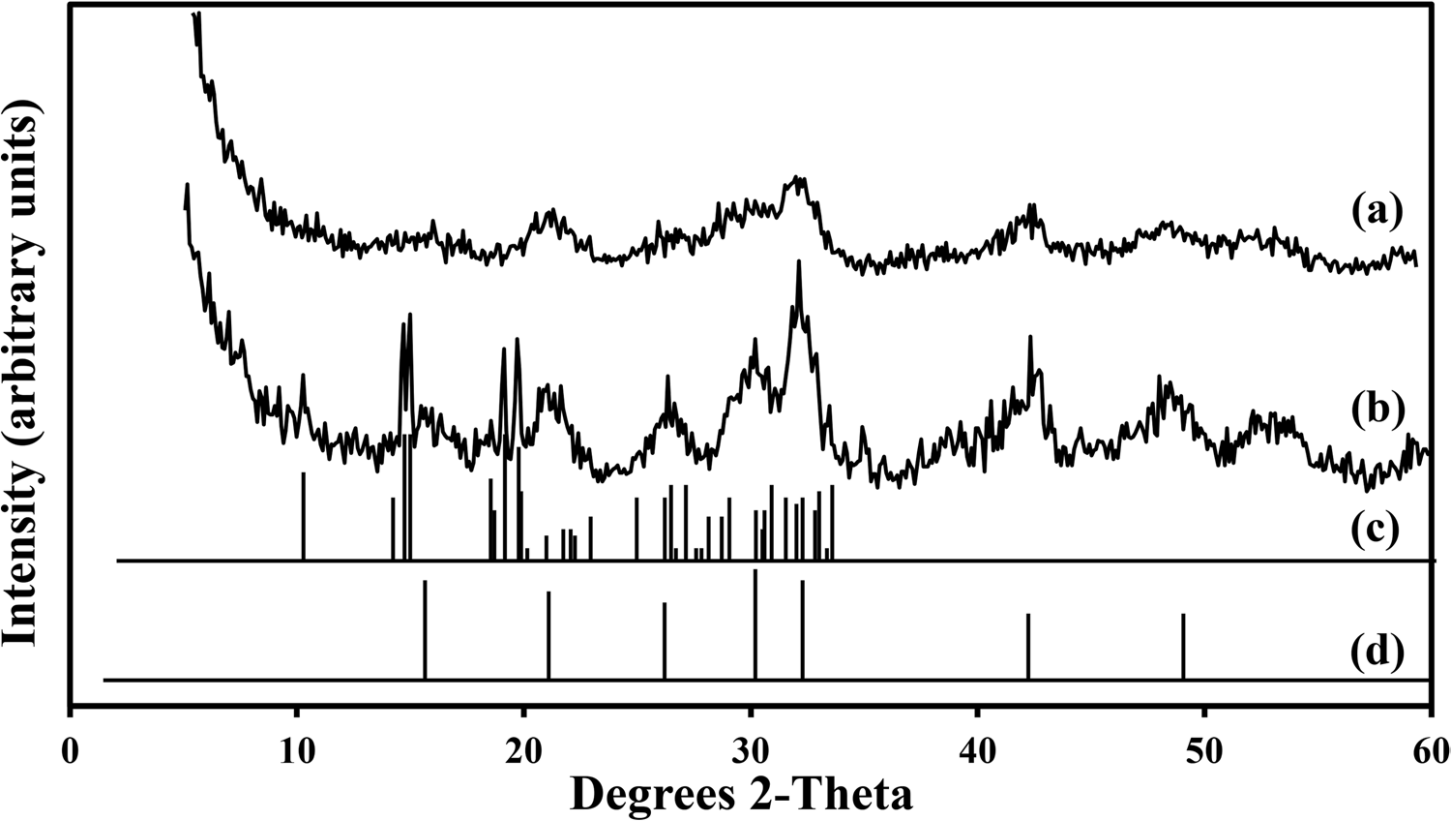
XRD patterns of the minerals formed after mixing of natural struvite leachate with (**a**) 20 mM and (**b**) 50 mM CeCl_3_ for 24 h at room temperature. (**c**) Standard reference pattern of the mineral Ce_2_(C_2_O_4_)_3_·10H_2_O (PDF card number: 20–268). (**d**) Standard reference pattern of the mineral Ce(PO_4_)·H_2_O. Typical patterns are shown from one of several determinations

XRD showed that the patterns of all the tested minerals were similar with a high background noise. Peaks at 13.14, 22.54, 27.64, 27.84, 29.45, 30.49 and 30.75 degrees 2-theta were identified and assigned to standard reference patterns. XRD patterns of the biominerals resulting from mixing the biomass-free struvite leachate with 20 mM CeCl_3_ (Fig. 6a) exhibited similarities with a match to only Ce(PO_4_)·H_2_O (Mooney 1950; Barrera-Villatoro et al. 2017) (Fig. 6d). The patterns of the biominerals formed by mixing the biomass-free struvite leachate with 50 mM CeCl_3_ (Fig. 6b) exhibited distinguishable peaks with a match to standard Ce(PO_4_)·H_2_O (Fig. 6d) and Ce_2_(C_2_O_4_)_3_·10H_2_O (Fig. 6c). Rietveld refinement analysis further revealed that the biominerals formed at 20 mM CeCl_3_ contained 100% Ce(PO_4_)·H_2_O (Table 2). The biominerals formed by mixing 50 mM CeCl_3_ with natural struvite leachate were composed of 78.3% Ce(PO_4_)·H_2_O and 21.7% Ce_2_(C_2_O_4_)_3_·10H_2_O.

**Table 2:** Quantification of the mineral phases in the samples through the Rietveld refinement of XRD data

Treatment		Mineral composition	
		**Ce(PO**_**4**_**)·H**_**2**_**O**	**Ce**_**2**_**(C**_**2**_**O**_**4**_**)**_**3**_**·10H**_**2**_**O**
Before TG-DTG	N + 20 mM Ce	100%	Undetected
	N + 50 mM Ce	78.3%	21.7%
		**CePO**_**4**_	**CeO**_**2**_
After TG-DTG	N + 20 mM Ce	100%	Undetected
	N + 50 mM Ce	90.4%	9.6%

N = biomass-free natural struvite leachate

Typical values are shown from one of several determinations

**Fig. 7:**
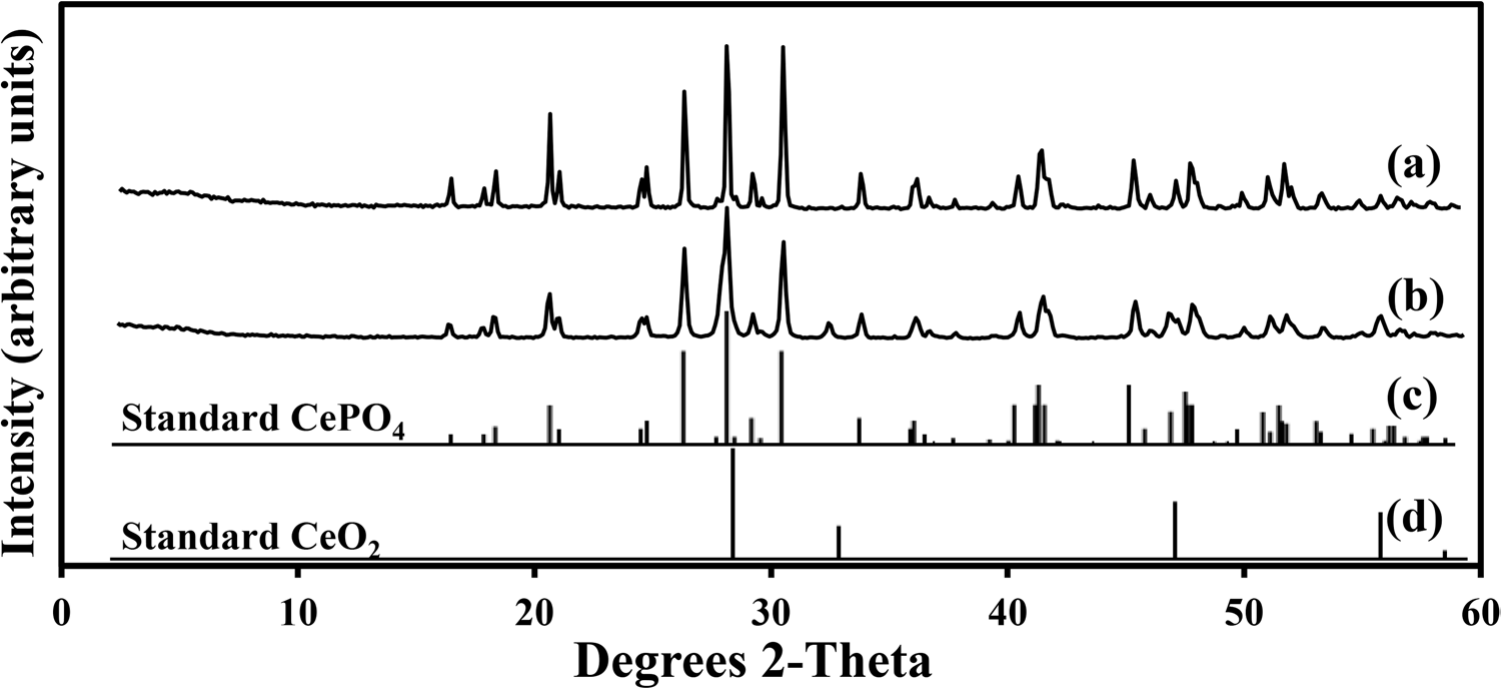
XRD patterns of the minerals after TG-DTG thermal treatment at 1000 °C. The minerals were formed after mixing CeCl_3_ with natural struvite leachate for 24 h at room temperature. (**a**) Natural struvite leachate with 20 mM CeCl_3_. (**b**) Natural struvite leachate with 50 mM CeCl_3_. (**c**) Standard reference pattern of the mineral CePO_4_ (PDF card number: 32–199). (**d**) Standard reference pattern of the mineral CeO_2_ (PDF card number: 34–394). Typical patterns are shown from one of several determinations

After thermal treatment at 1000 °C, minerals precipitated at both concentrations showed improved crystallinity with sharp peaks and a very low noise base (Fig. 7a and b). The biomineral sample resulting from mixing natural struvite leachate with 20 mM CeCl_3_ showed a match to standard CePO_4_ (Fig. 7c), while that obtained from mixing natural struvite leachate with 50 mM CeCl_3_ comprised CePO_4_ and CeO_2_ (cerianite) (Fig. 7d). Rietveld refinement analysis revealed that the biominerals precipitated using natural struvite leachate at 20 mM CeCl_3_ contained 100% CePO_4_ (Table 2). The sample formed using natural struvite leachate at 50 mM CeCl_3_ was composed of 90.4% CePO_4_ and 9.6% CeO_2_ (Table 2).

**Fig. 8:**
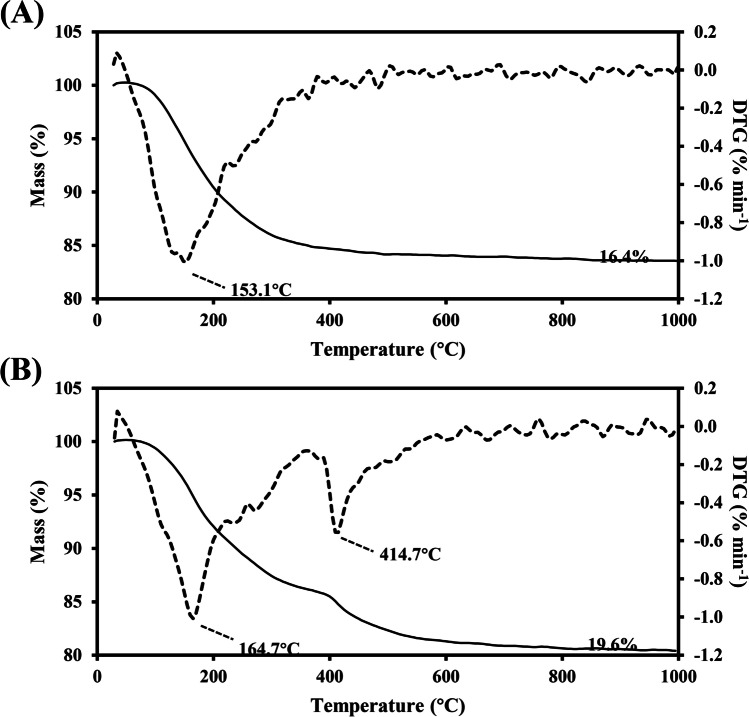
Thermogravimetric analysis of the biominerals formed by mixing natural struvite leachate with (**A**) 20 mM and (**B**) 50 mM CeCl_3_ for 24 h under room temperature. Continuous line: TG curves. Broken line: DTG curves. Typical curves are shown from one of several determinations

Thermogravimetric analysis (TGA) showed that the biomineral samples precipitated at 20 mM CeCl_3_ using the natural struvite leachate had a 16.4% mass-loss event at 153.1 °C according to the thermogravimetry (TG) and derivative thermogravimetry (DTG) curves (Fig. 8A). The DTG curve showed two distinct thermal events at 164.7 °C and 414.7 °C with a total mass-loss of 19.6% for the biomineral sample formed by the precipitation of natural struvite leachate at 50 mM CeCl_3_ (Fig. 8B).

**Fig. 9:**
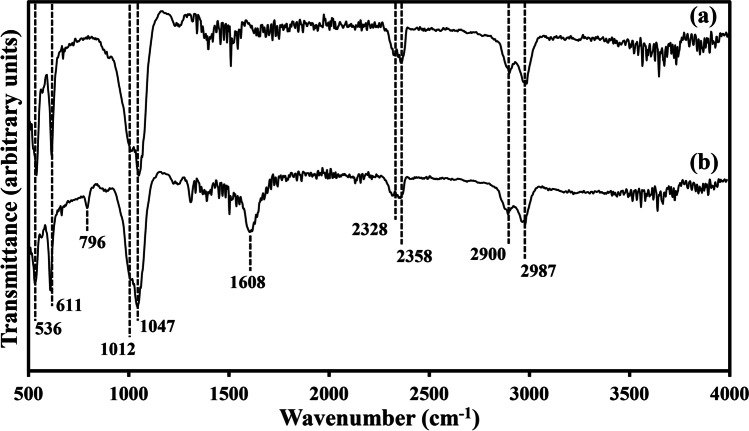
FTIR spectra of the samples resulting from the reaction of (**a**) 5 and 20 mM CeCl_3_ with natural struvite leachate, (**b**) 40 and 50 mM CeCl_3_ with natural struvite leachate. Typical spectra are shown from several similar determinations

The amorphous biominerals resulting from the reaction of struvite leachate with 5, 20, 40 and 50 mM CeCl_3_ were further analyzed using FTIR. Small and sharp peaks at around 536 and 611 cm^−1^ and broad peaks at around 1012 and 1047 cm^−1^ were observed in all samples tested (Fig. 9). Medium-sized peaks at 2328–2358 cm^−1^ and 2900–2987 cm^−1^ were observed for all biomineral samples (Fig. 9). A minute peak at 796 cm^−1^ and a medium-sized peak at 1608 cm^−1^ were observable in only the biomineral samples precipitated at higher Ce concentrations (40 and 50 mM CeCl_3_) (Fig. 9b).

## Discussion

The natural struvite sample contains a rich source of P, which was completely released after incubation with *A. niger* in liquid MCD medium. The complete dissolution of struvite was caused by acidolysis resulting from fungal-produced oxalic acid (H_2_C_2_O_4_) (Suyamud et al. 2020; Ferrier et al. 2021). Compared to the struvite-free control, lower amounts of oxalic acid in the struvite-containing groups were due to the consumption of oxalic acid, which can result in the formation of insoluble magnesium oxalate. Interestingly, *A. niger* yielded significantly more biomass and exhibited more luxuriant growth in the medium with natural struvite than without struvite. The growth state of *A. niger* can be affected by nitrogen and phosphorus source, and the presence of trace elements, which contribute greatly to the normal functioning of physiological processes in organisms (Agnihotri 1967). The natural struvite sample contains ammonium from sewage pipes (Kecskésová et al. 2020), which provides an extra nitrogen source for mycelial growth (Gadd 2007). MCD medium only contains basic nutrients and was devoid of trace elements such as Cu, Mn and Zn. Besides Mg and P, the natural struvite sample contains Ca, Co, Fe and Mn, all of which are important trace elements. In other work, *A. niger* yielded 25% more biomass in a struvite-free AP1 medium, which contained trace elements, than in a medium amended with 1% natural and synthetic struvite (Suyamud et al. 2020). Therefore, it appears the presence of ammonium and trace elements in the natural struvite also contributed to the luxuriant growth of *A. niger*. Moreover, the use of medium with minimal components can reduce impurities in the resulting biominerals, which is important for biorecovery purposes.

The quantification experiments showed that more than 99% of Ce^3+^ was removed from solution and a large amount of precipitate was formed. The significant decline of pH was due to the hydrolysis of Ce^3+^ (Xue et al. 2017) as excessive amounts of CeCl_3_ were mixed with the biomass-free culture medium. The biominerals precipitated contained a high amount of P and appeared to be nanoscale amorphous materials with no distinguishable boundaries. Because of this, Rietveld refinement was adopted in association with XRD for a more accurate determination of the mineral phases contained in the materials. The XRD-Rietveld results indicated that small amounts of oxalates were formed at a higher Ce^3+^ concentration (50 mM CeCl_3_), which was corroborated by the SEM images that showed large oxalate-like crystals amid an amorphous background at this concentration, thus confirming the occurrence of cerium oxalate decahydrate [Ce_2_(C_2_O_4_)_3_·10H_2_O]. This also indicated that the Ce phosphate [Ce(PO_4_)·H_2_O] biominerals, formed at 20 mM CeCl_3_ using the biomass-free natural struvite culture medium and subsequently transformed into 100% CePO_4_, were of high purity and high thermal stability. The presence of CeO_2_ in the samples after thermal treatment was due to the thermal decomposition of Ce_2_(C_2_O_4_)_3_·10H_2_O formed at 50 mM CeCl_3_. The resulting Na_3_Ce(PO_4_)_2_ in the samples after calcination probably arose from Na-containing impurities in the biominerals. One study discovered that β-Na_3_Ce(PO_4_)_2_ was an intermediate compound in a CePO_4_-Na_3_PO_4_ hydrothermal crystallization system at 950 °C (Xu et al. 1996). The TG-DTG curves corroborated XRD-Rietveld results in that there was single component in the biominerals precipitated using the natural struvite leachate at 20 mM CeCl_3_. The mass-loss at 153.1 °C (precipitation of natural struvite leachate with 20 mM CeCl_3_) can be attributed to the evaporation of water and the dehydration of water of crystallization in Ce(PO_4_)·H_2_O. In the biomineral sample formed using the struvite leachate at 50 mM CeCl_3_, two thermal decomposition events were recorded, i.e. the one at 164.7 °C was attributed to the loss of water and the other at 414.7 °C to the transformation of Ce_2_(C_2_O_4_)_3_·10H_2_O into CeO_2_.

FTIR spectroscopy was employed for better identification of functional groups in the amorphous minerals. The characteristic peaks at 536 and 611 cm^−1^ were due to the *v*_4_ vibrations of O–P–O bonds (Masui et al. 2003; Li et al. 2018). The absorption band around the strong dual peaks at 1012 and 1047 cm^−1^ was attributed to the *v*_3_ vibration modes of O–P–O bonds (Tie et al. 1997; Li et al. 2016). The most conspicuous spectral difference was that the experimental samples showed distinctive peaks at around 2328–2358 cm^−1^ and 2900–2987 cm^−1^. The peak around 2328 and 2358 cm^−1^ is characteristic of O = C = O asymmetric stretching (Bal et al. 2001). The medium-sized band around the double peaks at 2900 and 2987 cm^−1^ was due to -CH_2_ and -CH_3_ (symmetric and asymmetric) stretching from aliphatic groups, possibly arising from saturated fatty acids, lipids and proteins (Georgakopoulos 2003; Lazar et al. 2012; Mecozzi et al. 2012). Similar double-peak patterns over this absorption range were shown for EPS-associated jarosite [KFe^3+^_3_(OH)_6_(SO_4_)_2_], which was biosynthesized by *Purpureocillium lilacinum* (Bao et al. 2018). The moderate absorption around 1608 cm^−1^ for biominerals formed at 40 and 50 mM CeCl_3_ is assigned to the C = O stretching of oxalate (Pan and Wang 2017), which again corroborated the XRD-Rietveld results that cerium oxalate was formed at higher Ce concentrations. The FTIR results also implied that the amorphous biominerals were probably precipitated in association with organic matter, as it has been reported that extracellular polymeric substances produced by *A. niger* and *Aureobasidium pullulans* contain tryptophan-like and aromatic protein-like substances and can have interactions with Co, Hg, Ni and Se (Yang et al. 2019; Song et al. 2020).

Our results provide a novel approach to the treatment of naturally occurring struvite by fungal-produced oxalic acid. The resulting leachate was able to remove Ce^3+^ leading to the formation of amorphous biominerals, which were identified as cerium phosphate [Ce(PO_4_)·H_2_O] at low Ce concentration and a mixture of cerium phosphate and cerium oxalate [Ce_2_(C_2_O_4_)_3_·10H_2_O] at a high Ce concentration. The phosphate-containing biominerals formed in the natural struvite leachate at 20 mM CeCl_3_ showed high purity and excellent thermal stability and can be transformed into CePO_4_ after thermal treatment. This study has provided insights into the biomineralization of REE by fungal-mediated oxalate and phosphate precipitation systems, which have a potential for metal biorecovery, bioremediation and biosynthesis of nanomaterials.
